# Death from Cancer and Cardiovascular Disease between Two Brazils

**DOI:** 10.36660/abc.20200017

**Published:** 2020-02

**Authors:** Sílvia Marinho Martins

**Affiliations:** 1 Pronto Socorro Cardiológico de Pernambuco (PROCAPE), Ambulatório de Doença de Dhagas e Insuficiência Cardíaca, Recife, PE - Brazil; 2 Real Hospital Portugues de Beneficencia em Pernambuco (Realcor), Recife, PE - Brazil

**Keywords:** Cardiovascular Diseases/mortality, Coronary Artery Disease/physiopathology, Neoplasms/mortality, Epidemiology

## Introduction

Population aging has been represented as one of the main global trends in future prospects, and Brazil is increasingly becoming established within this scenario. While life expectancy is estimated to be over 83 years in countries like Japan, Switzerland, and Spain, in others such as Nigeria and Somalia individuals reach an average age of 55. In 2018, life expectancy was estimated at 71 years in Brazil.^1^ The relationship between health and development is quite complex, and it presents countless interactions. Both life expectancy and main causes of death appear as indicators of a region or a country’s quality of life. They are signs of lifestyle (and advice regarding adequate change), preventative healthcare services provided to the community and advances in diagnostic techniques.^2^ Health conditions are influenced by the socio-economic environment, given that higher indicators of income and educational level manifest as adoption of healthier lifestyle habits and, naturally, access to more effective treatment.^3^

In the past, communicable diseases represented the leading cause of death. In low-income countries, 52% of deaths were caused by communicable diseases, conditions resulting from pregnancy and childbirth, and other issues related to nutritional deficiencies. In contrast, in high-income countries, these causes accounted for at most 7% of deaths.^4^ It is estimated that, by 2030, most countries will have made the much acclaimed epidemiological transition and have profiles with a higher prevalence of non-communicable diseases.

### Relationship between economic development and cardiovascular mortality

In 2016, of the 56.9 million deaths that occurred worldwide, ischemic heart disease and stroke were the two leading causes, and they have remained the main causes of global death over the past 15 years. It is, nonetheless, worth emphasizing that causes of mortality vary according to countries’ wealth patterns.^4^

A recent publication presents data from a prospective, multicenter study involving 155,722 participants from 21 different countries, which evaluated risk factors and mortality from cardiovascular diseases (CVD).

Countries were stratified according to level of economic development. It was observed that the majority of diseases and deaths related to the cardiovascular system could be attributed to a small number of modifiable risk factors, some with important effects, others varying by the countries’ economic levels. The study emphasizes, moreover, that health policies should concentrate on specific risk factors. For example, low educational level’s association with CVD and death was strongly identified in countries with low to medium economic development. In developed countries, 70% of CVD were attributed to modifiable risk factors (with the exception of environmental pollution), with the important contribution of metabolic risk factors and tobacco use. In undeveloped countries, 80% of diseases and deaths due to cardiovascular etiology were attributed to modifiable risk factors, with the important contribution of metabolic factors, environmental pollution, and poor diet. Level of education’s association with death is even stronger than its association with wealth. From early childhood onward, education affects multiple living conditions, including living and working in healthier environments and greater access to health services. It is worth reiterating that improvements in education will likely decrease the number of deaths from different conditions, indicating that investments in this area could bring wide reaching benefits to health.^5^

### Socioeconomic aspects of the incidence of malignant neoplasms

The prevalence of cancer is relevant worldwide. In 2018, on the global level, one in every six deaths was related to this group of diseases. Malignant neoplasms are also responsible for approximately 70% of deaths in low- and middle-income countries.^6^

Some Western countries have managed to control the incidence of determined types of cancer by reducing the prevalence of classic risk factors, as well as by early detection and appropriate treatment. However, lung, breast, and cervical neoplasms continue to increase significantly, due to risk factors typical in Western countries, such as tobacco use, obesity, sedentarism, and changing reproductive patterns. Organs such as the stomach, liver, and cervix also continue to present high morbidity related to infection.

Countries with high economic development continue to present high incidences of lung, colorectal, breast, and prostate cancer. What are distinct, however, are the mortality rates; while there is a reduction in the number of deaths from cancer in developed countries, this figure increases in undeveloped countries. Mortality rates due to malignant neoplasms have been increasing in countries with low levels of development, as a consequence of increased prevalence of risk factors, such as increased tobacco use, excess body weight, physical inactivity, and better treatment.^7^

The article “Trends in Mortality Rates from Cardiovascular Disease and Cancer between 2000 and 2015 in the Most Populous Capital Cities of the Five Regions of Brazil”^8^ presents important conclusions regarding the incidence of cancer and CVD in relation to a country’s level of development. In England, for instance, the rate of mortality from CVD decreased more than the rate from cancer. In individuals over the age of 75, in particular, the impact of advances made in diagnosis and treatment on the mortality rate from cancer was even lower. In 2011, the age-standardized mortality rate from cancer exceeded that of CVD in both sexes, in the United Kingdom, long before this is observed in Brazil.^9^

In the USA, mortality rates from cancer in adults between the ages of 45 and 64 decreased by 19%, from 1999 to 2017, whereas rates of mortality from heart diseases decreased by 22%, from 1999 to 2011, and then increased by 4% from 2011 to 2017. The mortality rate from cancer has always been higher than that from heart diseases in that country.^10^

## Final considerations

In this manner, Brazil continues with its demographic transformation and its epidemiological transition. Population aging, as previously observed, is a foreseeable trend, allowing society and individuals to plan for this new profile. As a national panorama, our demographic profile is already similar to that of large countries. The elderly group (individuals over the age of 60) is growing faster than any other age group in Brazil. Nevertheless, the epidemiological transition continues with inequality: The group of Brazilians who live with housing and income conditions similar to those in developed countries also demonstrate morbidity and mortality similar to those countries, whereas the rest of the population, which constitutes the majority of Brazilians, lives in poverty with scarce healthcare resources.

A correlation was recently demonstrated between the evolutionary variation of gross domestic product (GDP) per capita in the municipalities of the state of Rio de Janeiro and reduced mortality from coronary artery disease.^11^ This once again emphasizes the importance of the need for better living conditions in order to reduce cardiovascular mortality. For this and other reasons, Martins et al.^8^ draw attention to the need to fragment the discussion regarding death trends in Brazil, due to the distinct profiles the country presents, which correspond to different GDP and indicators of education.

The main challenge in Brazil will be that of recognizing this new profile that is being unveiled and designing targeted strategies according to the peculiarities found, leading to greater health promotion for the entire population in an equitable manner.
